# Rigid–Soft Interactive Design of a Lobster-Inspired Finger Surface for Enhanced Grasping Underwater

**DOI:** 10.3389/frobt.2021.787187

**Published:** 2021-12-22

**Authors:** Haiyang Jiang, Xudong Han, Yonglin Jing, Ning Guo, Fang Wan, Chaoyang Song

**Affiliations:** ^1^ Department of Mechanical and Energy Engineering, Southern University of Science and Technology, Shenzhen, China; ^2^ AncoraSpring, Inc., Shenzhen, China

**Keywords:** soft robotics, robotic grasping, underwater manipulation, biomimetics, rigid–soft interaction

## Abstract

Bio-inspirations from soft-bodied animals provide a rich design source for soft robots, yet limited literature explored the potential enhancement from rigid-bodied ones. This paper draws inspiration from the tooth profiles of the rigid claws of the Boston Lobster, aiming at an enhanced soft finger surface for underwater grasping using an iterative design process. The lobsters distinguish themselves from other marine animals with a pair of claws capable of dexterous object manipulation both on land and underwater. We proposed a 3-stage design iteration process that involves raw imitation, design parametric exploration, and bionic parametric exploitation on the original tooth profiles on the claws of the Boston Lobster. Eventually, 7 finger surface designs were generated and fabricated with soft silicone. We validated each design stage through many vision-based robotic grasping attempts against selected objects from the Evolved Grasping Analysis Dataset (EGAD). Over 14,000 grasp attempts were accumulated on land (71.4%) and underwater (28.6%), where we selected the optimal design through an on-land experiment and further tested its capability underwater. As a result, we observed an 18.2% improvement in grasping success rate at most from a resultant bionic finger surface design, compared with those without the surface, and a 10.4% improvement at most compared with the validation design from the previous literature. Results from this paper are relevant and consistent with the bioresearch earlier in 1911, showing the value of bionics. The results indicate the capability and competence of the optimal bionic finger surface design in an amphibious environment, which can contribute to future research in enhanced underwater grasping using soft robots.

## 1 Introduction

The biological structure has been a critical source of design inspiration for advanced robotic systems, where soft-bodied animals, both on land and under the water, have shown numerous successful applications in novel designs of soft robots (Stilli et al., 2014; Te et al., 2020; Mao et al., 2013). The organic nature of natural forms usually involves a mixture of rigid and soft components, not only for the basic survival of the living organism but also as a crucial factor while interacting with the physical world. Endoskeletons, including that of humans, usually incorporate the musculoskeletal system for motion generation and torque control, a range of sensory feedback to provide diverse tactile information to aid the decision making, as well as the skin layer to provide the necessary frictional interaction with the target object or environment for a successful integration (Tavakoli et al., 2017). However, there are also a class of creatures with exoskeletons, where the rigid shell structure encloses the soft muscles and organs as a result of natural selection, commonly observed among the crustaceans that live under the ocean or in an amphibian environment with both land and water (Xu et al., 2020). In this case, the tactile information remains available but is restricted to a limited extent. It becomes an exciting subject to study the “skin” pattern of the rigid shells among these exoskeleton animals during manipulation tasks while operating in a challenging environment with water, which is a field with limited research.

The ocean is critical to life on Earth, which covers over 70% of our planet, and nearly 40% of the population of the world live within 60 miles of the sea (Merz, 2001). While most animals under the ocean, such as various breeds of fish, use mouth and whole-body motion for effective manipulation, some animals such as the crustaceans, including lobsters, crayfish, and crabs, and Cephalopoda, including cuttlefish, and octopus, have evolved arms or tentacles with dexterous skills for object manipulation under the water. Recent research on soft robotics explored the bio-inspired design from the Cephalopoda, where the whole-body softness enables a new class of robot design, modeling, and control for object manipulation. For example, Stilli et al. (2014) explores a new hybrid actuation principle combining pneumatic and tendon-driven actuators for a soft robotic manipulator taking inspiration from the octopus. Limited research has explored the adoption of biological features from the lobsters for novel robot systems. Earlier research by J. Ayers explored the neurobiological patterns from the lobster limbs while walking stealthily on the seabed, including a series of prototypes mimicking the gait pattern and mechanism of the lobster (Ayers and Crisman, 1992). Recent research also explored the design integration of external rigid shells and soft fluidic actuators inside for more efficient actuation with the ease of kinematic modeling (Chen et al., 2017a) (Chen et al., 2017b) as well as volumetrically-enhanced performance (Wang et al., 2021).

While much research on object manipulation has been devoted to the efficient and effective grasping problems on land, it remains a challenging subject to study effective grasping under the water, where 3 major design approaches were explored in the literature, where under-actuated systems are the common choice to balance design complexity and system performance for underwater usage.

• Direct translation of rigid mechanisms from on-land to underwater purposes was the main approach in earlier designs, which usually adopted common gripper mechanisms for on-land use and then redesigned with complex waterproofing for underwater scenarios (Licht et al., 2016). Such a direct translation of technology becomes challenging to integrate the various sensors, which are usually common for on-land use, as the targeted working depth goes deeper (Takeuchi et al., 2018).
• Cable-driven system is another approach to relocate the actuator for effective waterproofing while enabling an effect and compliant grasping underwater. For example, the second and third generation of the Bolonia hand (Ribas et al., 2015), the 3-finger gripper on Stanford’s OceanOne (Brantner and Khatib, 2021), as well as the waterproofed version of the Pisa-IIT Soft Hand all adopted the cable-driven tendon system for underwater grasping with compliant adaptation, where a series of sensors were integrated for intelligent grasping underwater.
• Soft robot approach recently emerged as an alternative method with a significant reduction in system complexity while leveraging the fluidic environment under the water for depth-invariant actuation. Harvard University proposed a gripper design by integrating the PneuNet structure with fiber-reinforced design and used memory foam as the finger surface for fragile life form grasping at a depth of 2,000 m under the water (Galloway et al., 2016). Researchers from the Beihang University also adopted the PneuNet design but used it for a soft robotic arm for underwater use (Gong et al., 2021). Recent research also explored a hybrid design with soft actuators and a rigid gripper mechanism for depth-invariant grasping. However, there remains a limited adoption of sensory capabilities with soft underwater robots, mainly due to a lack of research as an emerging field.

**FIGURE 1:**
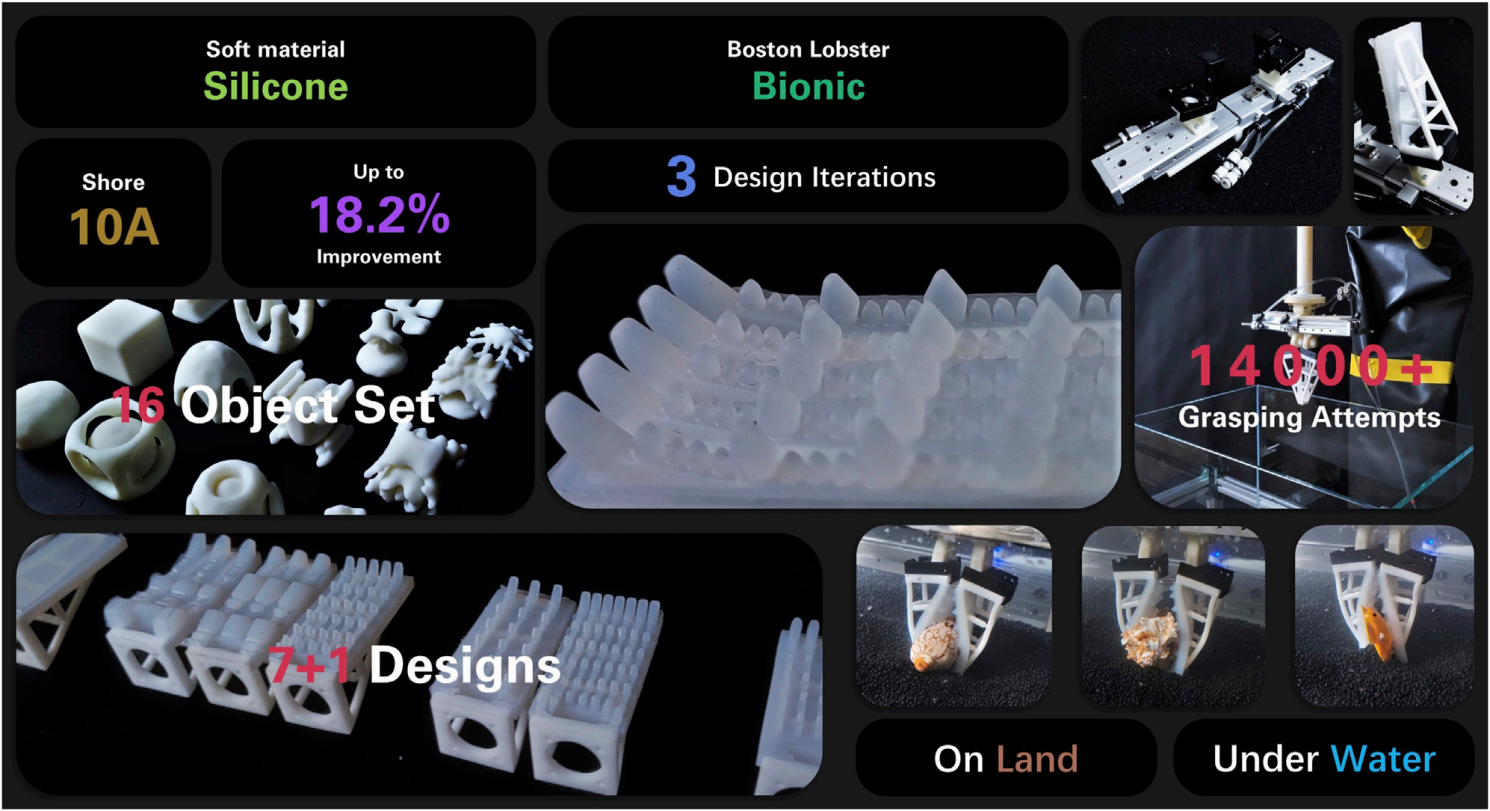
Inspired by the Boston Lobster, we designed 7 bionic finger surface patterns in 3 iterations and fabricated them using silicone of Shore 10A. The designs are attached to the soft adaptive fingers and are tested on a set of 16 challenging objects. The optimal design is selected through over 14,000 grasp attempts, proves its capability and competence not only on land, but also underwater, and shows an improvement of up to 18.2%.

In this paper, we adopted various tooth profiles from the rigid claws of the Boston Lobster, as shown in Figure 1, to design a series of robotic finger surface patterns that are found to provide effective enhancement for grasping underwater. These finger surface patterns were fabricated by a soft material, which is not identified with the original rigid claw of the lobsters. Classical bio-inspired design usually adopts direct mimicking of the natural features of the animal. In this paper, we proposed a different approach by looking at the rigid–soft interaction for design inspirations of our gripper from on-land use to underwater grasping. For example, lobsters with rigid shells usually prey on soft-muscled animals, such as fish, for feeding. However, this is usually the opposite case with robotic grasping, which commonly deals with rigid objects in engineering applications, where a soft gripper can become a potential solution to reproduce a rigid–soft interaction during grasping. Our previous research shows promising results by adopting such design strategy for efficient grasping learning (Yang et al., 2020). In this paper, we intended to draw inspiration from the tooth patterns of the lobster claws, aiming at an enhanced grasping performance with a bio-inspired soft finger surface design and integration, which motivates our research presented in this paper.

**FIGURE 2:**
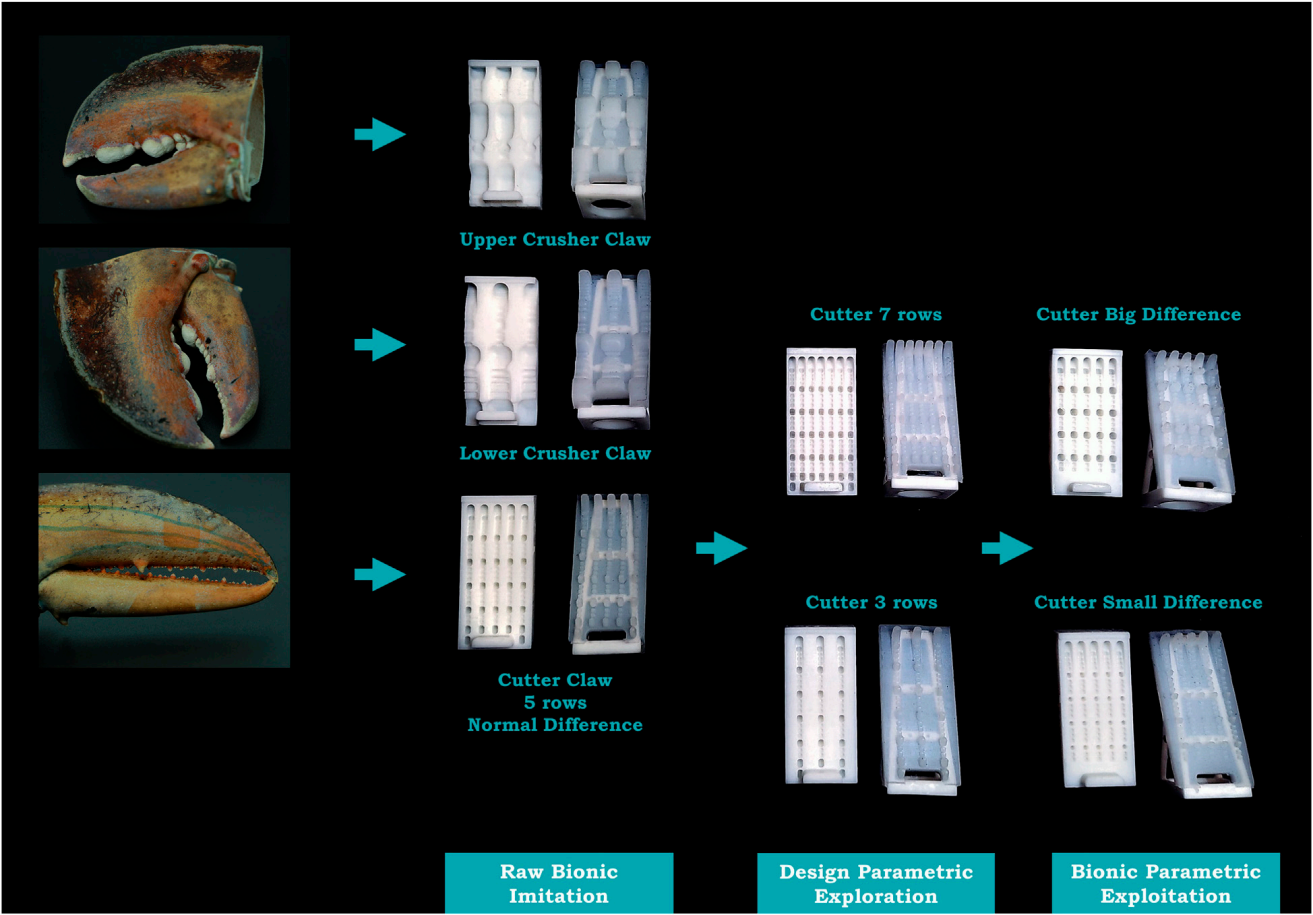
Design iterations process used in this paper, including raw imitation, design parametric exploration, and bionic parametric exploitation, based on bio-inspiration from the rigid tooth profiles of the lobster, to the various soft finger surface designs used in this paper.

Using a vision-based grasping system and evaluation method, we developed in our earlier research for on-land use (Jiang et al., 2021), we adopted the iterative design process that involves raw imitation, design parametric exploration, and bionic parametric exploitation to enrich the bionic design pool as shown in Figure 2. The contributions of this paper are as follows:

• We explored a lobster-inspired bionic design for a soft robotic finger surface by leveraging the rigid–soft interactions for both on-land and underwater usage.
• Our results provide the statistical evidence for the effectiveness of grasping underwater with the tooth profile of the lobster even fabricated with soft materials.
• The final optimal design provides positive enhancement for object grasping while integrated with a soft robotic finger with omni-directional adaptation for both on-land and underwater usage.

The rest of the paper is organized as follows. In the *Methods* section, we present the design inspiration from the rigid tooth profile of the lobster claw and set up the experiment environment and process for the following research. The *Results* section presents the detailed design iteration process about the progressive results for design enhancements. Discussions are enclosed in the *Discussion* section regarding the design translation from on-land to underwater usage, followed by the conclusion and limitations in the *Final Remarks* section.

## 2 Methods

### 2.1 Inspiration Drawn From the Rigid Tooth Profiles of the Lobster

Considered as “aesthetic ornament” without practical use in the very beginning, the lobster claws gradually proved their value as the research deepened, and eventually being recognized to be the most efficient tools and weapons for self-defense, capture of prey, rendering target in pieces, and handling over to the digestive system (Herrick, 1911). Each lobster is usually presented with 2 types of claws, namely, the cutter and the crusher. The cutter claw usually takes a slender shape with sharp and pointy edges on the tooth for cutting target in pieces, whereas the crusher claw is usually larger in size with big and rounded tooth for holding or breaking a target with a large force. Such combination is very similar to the use of fork and knife for human handling of food while eating. The teeth on the cutter are more like spines, while those on the crusher are more like tubercles. Besides, the teeth on a claw also differ from its propodus, which is the upper jaw, and dactyl, which is the lower jaw. The serration of the dactyl of the cutter is more regular than that of the propodus but still similar. However, the size (both length, width, and height) of the large tubercles on the propodus of the crusher are much larger than that on the dactyl. Last but not the least, thanks to the curved dactyl, the arrangement of teeth is different on the 2 jaws (Herrick, 1911).

**FIGURE 3:**
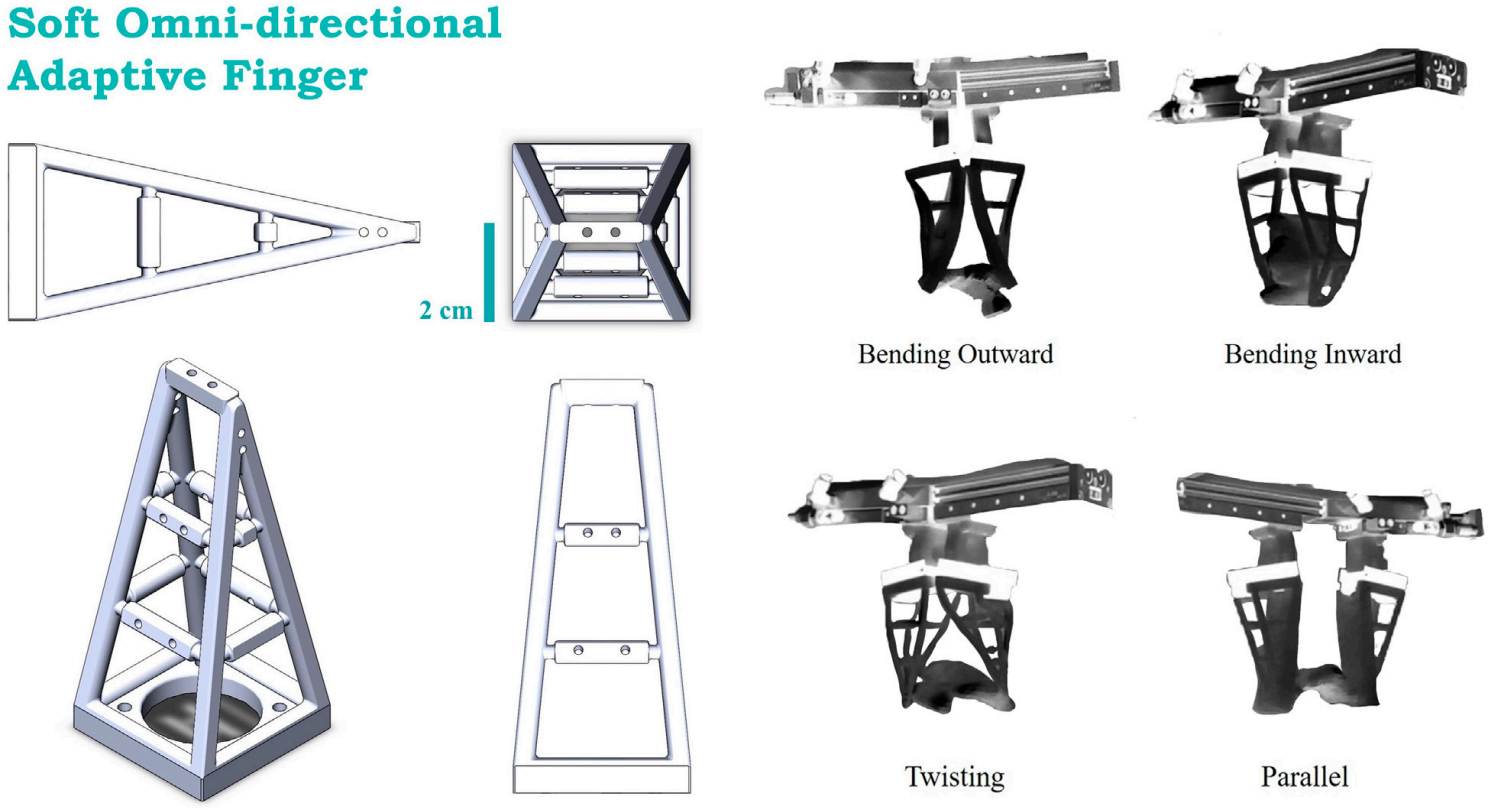
The execution unit that will be used in this paper, which is a network structure built by soft material, following with its 4 grasping poses.

We proposed a series of soft finger surfaces designs based on the different tooth profiles on the claws of lobster. To test their grasping performance, all the finger surface designs will be attached to the soft adaptive finger (Wan et al., 2020) shown in Figure 3. These soft fingers are adaptive in geometric form while interacting objects in 3D deformability. With this structure, the fingers can achieve a much enhanced form closure in 3 dimensions in grasping. Our aim is to test these surface designs along with the soft fingers empirically using a robotic manipulation system against selected objects from the Evolved Grasping Analysis Dataset (EGAD) (Morrison et al., 2020) to arrive at an optimal design with enhanced grasping reliability and stability.

### 2.2 Problem Formulation

While the soft, adaptive finger alone can conform to the geometric features of an object during the interaction, there are still ways to improve its grasping robustness. Due to the network design, the soft finger itself may not have sufficient contact area during grasping, causing grasp failures from time to time. This paper aimed to propose an integrated design with the finger surface to enhance the grasping robustness without interfering with its adaptive performance.

As Guo et al. (2017) have given detailed description and sufficient derivation of the problem finding the optimal design of the finger surface layer, we are describing our goal following their steps. We consider our goal as maximizing the likelihood of a successful grasping both on land and underwater. We define our design space to be *D*, and all of our designs follow *d* ∈ *D*. The object *o* to be grasped follows *o* ∈ *O*, where *O* is the test object set. Besides, it can be defined as *o* = (*u*, *v*), where $u\in R^{3}$ is the center of the object, and $v\in R^{3}$ is its orientation. Similarly, each single grasp can be defined as *g* = (*t*, *p*), where $t\in R^{3}$ is the grasp center of the gripper, and $p\in R^{3}$ is the orientation of the gripper. In this way, Γ = {(*g*_1_, *o*_1_), … (*g*_*n*_, *o*_*n*_)} is defined to be a set of experiment grasps and corresponding objects.

To label the success or failure of a grasp, we define *S*_*i*_(*d*) to be a binary variable recording the result of using design *d* to execute grasp *g*_*i*_ on object *o*_*i*_. “1” represents a success, while “0” represents a failure. Therefore, our goal becomes finding the optimal design that maximizes the mean likelihood for grasps on the formula:

$$d^{*}=\underset{d\in D}{\arg\max}\frac{1}{n}\overset{n}{\underset{i=1}{\sum }}P\left(S_{i}\left(d\right)=1\right),$$ (1)

where *n* is the expected number of successes. However, Equation 1 is abstract to deal with so that we further simplify the problem into maximizing the probability of success for the optimal design on grasps and objects. The probability of success is estimated by calculating the percentage of successes over *m* total grasp trails:

$$P\left(d,g_{i},o_{i}\right)=\frac{1}{m}\overset{m}{\underset{j=1}{\sum }}\left(S_{i,j}\left(d\right)=1\right),$$ (2)

where $S_{i,j}\left(d\right)$ is the *j*-th sample of $S_{i}\left(d\right)$ . Thus, our goal becomes choosing the design with the highest sample mean and lowest variance over the test object set to be the optimal design *d* ^∗^, which transforms a mathematical problem into an empirical-based problem.

### 2.3 A Robotic Manipulation Platform

**FIGURE 4:**
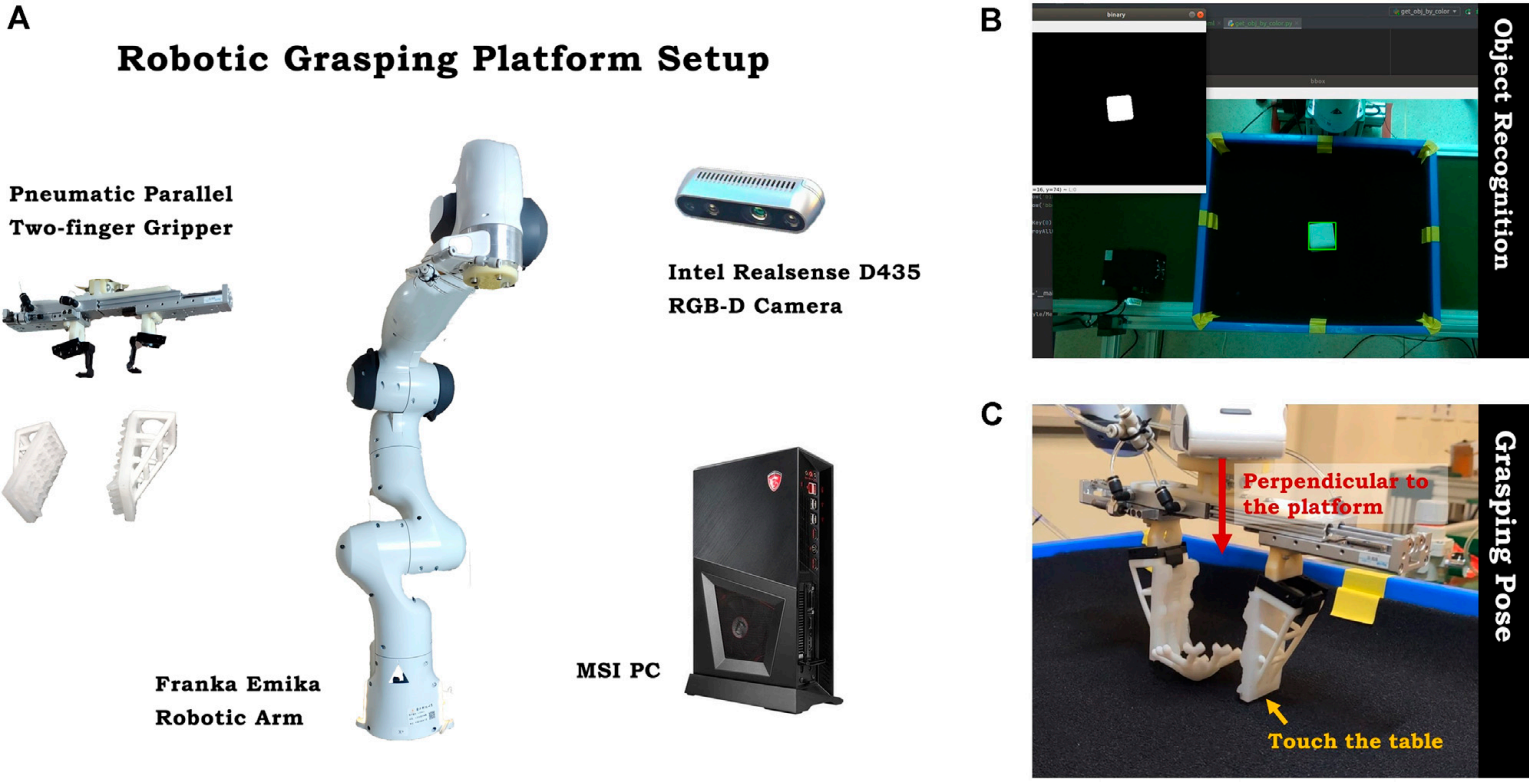
**(A)** The setup of the automatic robotic manipulation platform for the experiment. **(B)** Object recognition on the platform. **(C)** The perpendicular grasp pose for the gripper, with the fingers touching the surface of the platform.

Shown in Figure 4 is the robotic manipulation system used for testing, including a Franka Emika Panda as the robotic arm, a pneumatic gripper with parallel fingers as the gripper, a pair of soft, adaptive fingers for grasping, a PC from MSI as the computing and control unit, and a Realsense D435 as the RGBD camera. We used the pneumatic parallel gripper for both on-land and underwater usage. The gripper is powered by an air pump, with an air pressure of 1.0 MPa in a closing posture. The soft, adaptive fingers was mounted on the gripper. We used a layer of black foam on the surface of the table to avoid a collision, and for the underwater grasping, the tank is filled with 150-mm water depth for preliminary testing.

### 2.4 Grasping Position and Orientation for the Robotic Arm

The robotic arm is required to reach the grasp location first before it accomplishes a grasp action. The grasp location is (***x***, ***y***, ***z***, ***roll***, ***pitch***, ***yaw***). (***x***, ***y***, ***z***) is the 3D location in the Franka frame, while (***roll***, ***pitch***, ***yaw***) is the pose for the end-effector of the arm, with ***roll*** rotating about the ***x*** axis of the Franka frame, ***pitch*** rotating about the ***y*** axis, and ***yaw*** rotating about the ***z*** axis.

The center location of the object (***x***, ***y***) is calculated by an object recognition method called “color differentiating,” which is by putting a white object on the black background so that the camera can binary the image to recognize and bound the object in its frame (Figure 4B); ***z*** value is determined manually to let the fingertips touch the surface of the platform, the reason for this will be illustrated in a following experiment; (***roll***, ***pitch***, ***yaw***) value is (3.14, 0, ***angle***), which is a pose that the gripper is perpendicular toward the platform (Figure 4C), with ***angle*** value that can be arbitrarily rotating around the ***z*** axis. In this case, it is determined to be facing the computer.

### 2.5 A Challenging Object Set for Test

**FIGURE 5:**
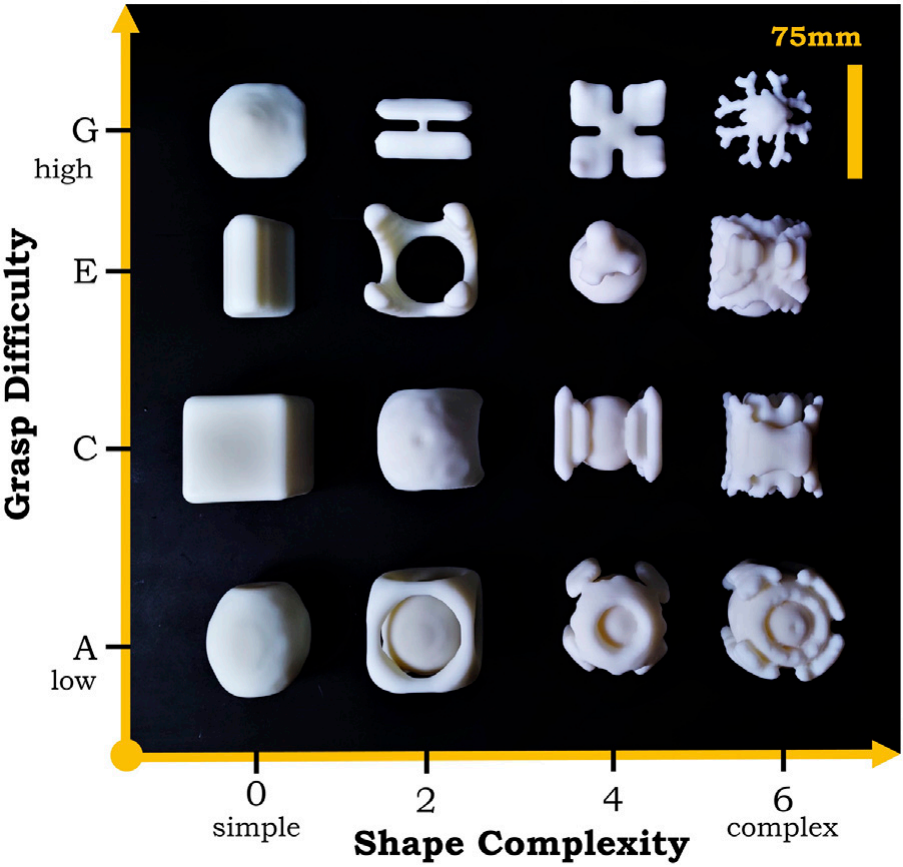
The 16 models selected from the Evolved Grasping Analysis Dataset (EGAD) that are challenging to pick in terms of grasp difficulty in the vertical axis and shape complexity in the horizontal axis. The models in this image are the specific 3D printed parts used in our experiments.

EGAD (Morrison et al., 2020) involves over 2,000 objects generated by an algorithm in terms of the grasp difficulty and shape complexity as the metric for how challenging they are in robotic manipulation. As shown in Figure 5, we selected 16 models out of the database with an even distribution on the scale and used them in our experiments to evaluate the performance of our finger surface designs. All objects are 3D printed with resin, and the material density is 1.12–1.18 g/cm^3^, which is slightly larger than water to ensure that they can sink to the bottom of the tank.

### 2.6 Experiment Process

Having decided the details above, we determined the workflow of a single grasp procedure in this experiment as:

• Step 1. Recognizing: The system will recognize the object in the grasping area and get its location.
• Step 2. Grasping: The system will command its robotic arm to reach the object and pressurize the air to close the fingers to grasp it.
• Step 3. Checking: The robotic arm will lift the object and move it outside the grasping area, and the camera will recognize the area again. If no object remains in the area, it indicates that the grasp is successful that the object has been removed. Otherwise, it indicates that the grasp fails and that the object has not been grasped or dropped by the fingers.
• Step 4. Releasing: as the check is finished, the robotic arm will move to the center of the grasping area and is 6.5 cm above the table surface. The robotic arm will release its gripper, letting the object freely fall to the table surface. This is supposed to give the object a random pose and location for the next grasping to simulate the uncertainty and noise in real-world situations.

The 4 steps above compose one single grasp, and each object will be grasped 80 times for each finger surface design until all the 16 test objects have been grasped, which means that each design is required to execute 16 × 80 = 1,280 grasps. We hoped a test result of this magnitude can reflect the true grasping capability of a design.

## 3 Results

### 3.1 Grasp at the Bottom Strategy

**FIGURE 6:**
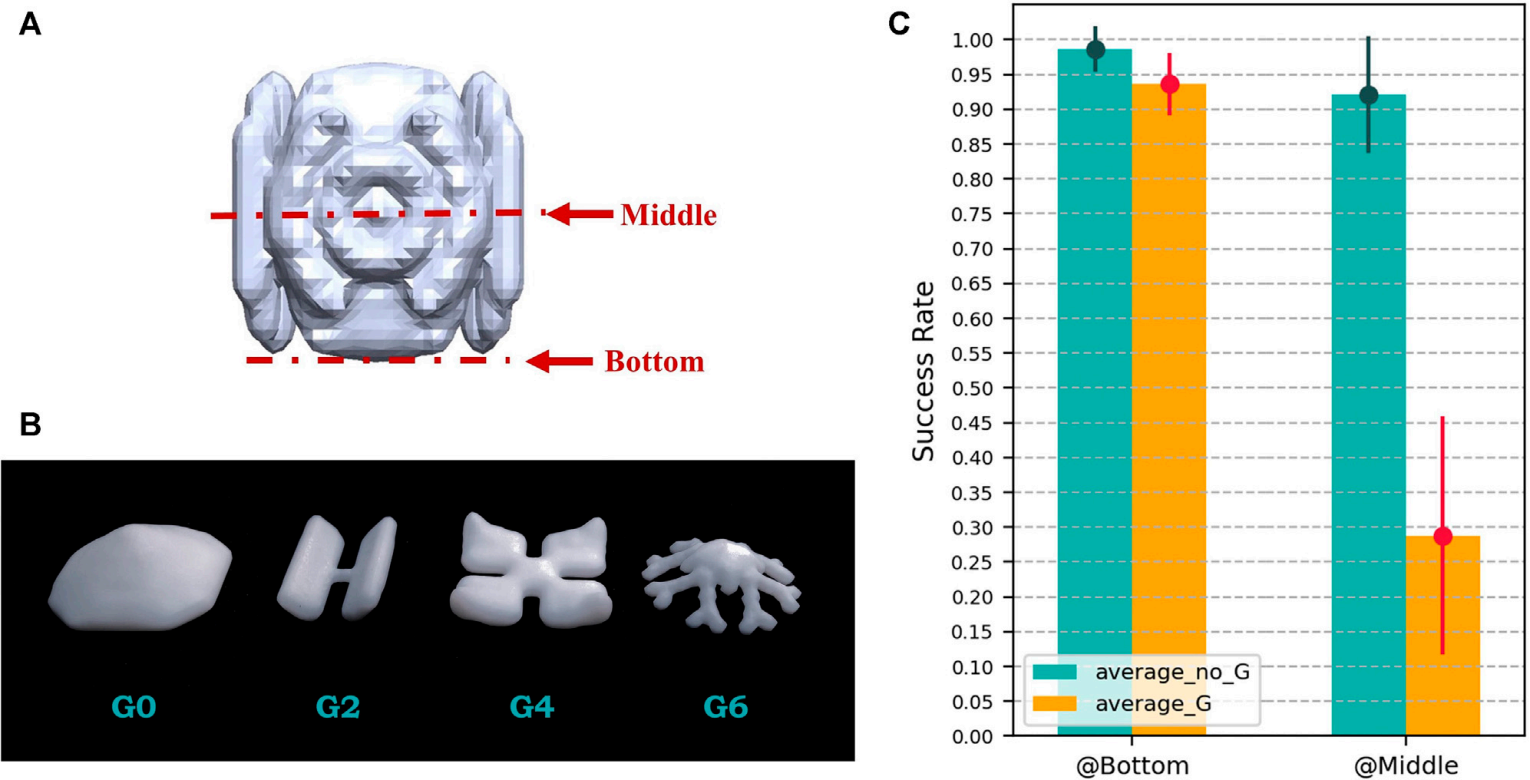
**(A)** Grasps at different positions of the object, middle and bottom. **(B)** “G” group objects, which are all of the flat shape, challenging for robotic grasping using the gripper, and are normally grasped using suction cup. **(C)** Grasping performance comparison between the grasping position at the bottom and middle of the object.

We set the *(z)* value to let the finger touch the surface of the platform, which is the so-called “grasp at the bottom strategy.” As shown in the Figure 6A, there are mainly 2 grasp positions: at the middle of the object, which is the commonly used position in the industry (Rodriguez et al., 2012), and at the bottom of the object, which requires the finger to be long enough to cover the whole object. We experimented by comparing which position can better benefit the grasping performance, using the cutter design to grasp on the test set, with the height of the object being measured manually. The result is shown in Figure 6C.

The result can be divided into 2 parts, the performance on group “G” objects (*average_G* in the bar graph) and the performance on objects without the “G” group (*average_no_G* in the bar graph). As shown in the Figure 6B, “G” objects are all of the flat shape, which provides limited areas for the finger to contact. In industrial solutions, these objects are manipulated using section cups since the flat surface provides enough area for the cups to suck (Mahler et al., 2019).

As the result shows, the strategy of grasping at the middle on “G” objects only yields a result of 28.8% with 17.1% variance, while the strategy of grasping at the bottom yields a result of 93.6% with 4.4% variance, holding a gap of an amazing 64.8%. As for objects without “G,” grasping at the bottom strategy holds a 6.5% lead on the mean and a 5.2% lead on the variance.

In a nutshell, grasping at the bottom is a more reliable and stable strategy than grasping at the middle. To utilize this strategy, 2 things should be ensured:

• The finger should be long enough to cover the whole object so that it can reach the bottom.
• Letting the finger touch the table surface might cause a collision, which will further cause the robotic arm to emergency stop and even result in security issues.

However, the latter problems can be solved by our soft, adaptive finger. Having the soft finger touch the surface will only cause the soft–rigid collision, generating a smaller force than the rigid–rigid collision and will not cause the robotic arm to an emergency stop. Therefore, thanks to the deformable soft material, this strategy can be safe for the soft finger. In this way, it can manipulate the challenging objects to the normal gripper with high reliability, which validates its universality on object manipulation.

### 3.2 First Iteration: Raw Imitation on the Boston Lobster Specimen

As mentioned in the *Introduction* section, we assumed that the secret for lobsters to accomplish daily manipulation tasks lies in the shape of its claw and the tooth profiles on them. Therefore, we observed and mimicked the tooth profiles of the lobster and transferred them into our contact surface layer design. We mimic the claws of Boston Lobsters for our soft finger design due to their similarity in size.

**FIGURE 7:**
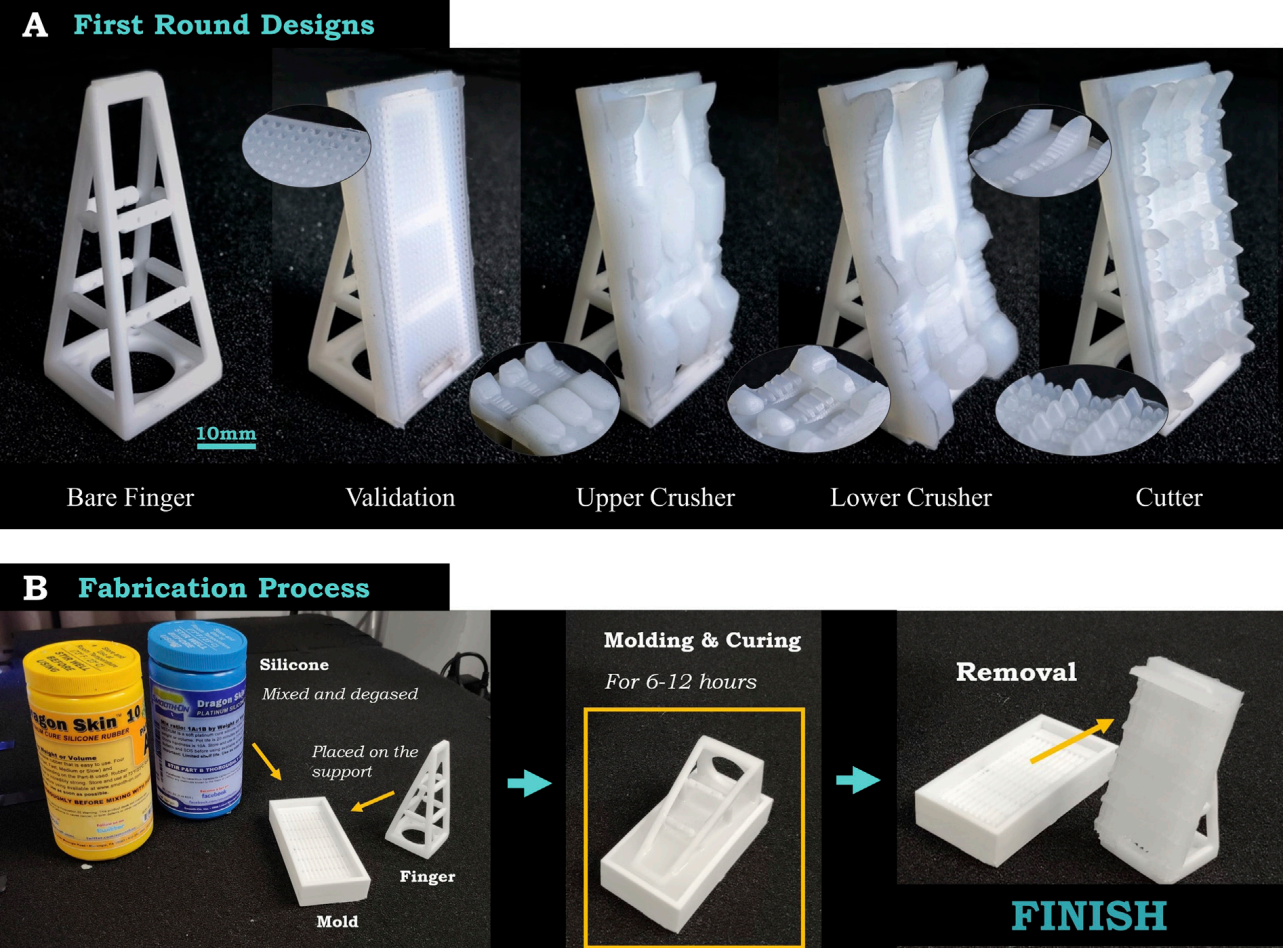
**(A)** The 5 design candidates tested in the first design iteration round, including 3 initial designs with finger surfaces, a bare finger design without finger surface and a pair of planar validation designs. These initial designs imitate the original shape of the claws of the Boston Lobster, and the validation design is from AUTOLAB at UC Berkeley in a rectangular rubber with multiple tiny voids on the surface. **(B)** The fabrication process to combine the molded silicone surface to the finger. Images are adapted from Figures 2,3,6 of (Jiang et al., 2021).

**FIGURE 8:**
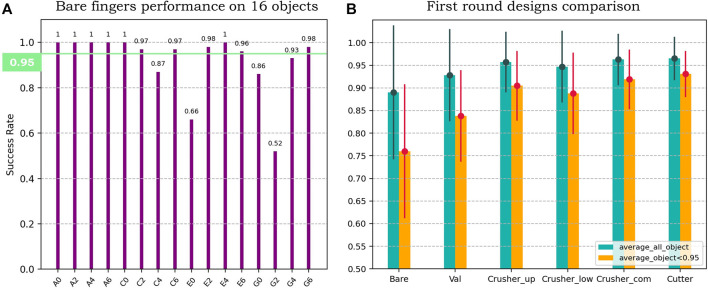
**(A)** The grasping result of the bare fingers on all 16 objects, with 11 objects higher than 95%. **(B)** The grasping result of the first round designs on the whole object set (green bars) and the shortlisted set (yellow bars), with mean and variance.

In the beginning, we came up with 3 initial designs by direct mimicking the claws. As shown in Figure 7A, “Upper Crusher” is a raw imitation of the upper crusher claw of the Boston Lobster, while “Lower Crusher” is of the lower crusher claw. Since these 2 parts hold dissimilarity to each other, they can be regarded as 2 different patterns. Besides, “Cutter” is a raw imitation of the cutter claw. The upper and lower parts of the cutter are similar to each other; thus, they are regarded as one pattern. Each pattern is duplicated into multiple rows to fill the whole surface area. Therefore, upper and lower crusher imitations only possess 3 rows due to their wider teeth, while cutter imitation possesses 5 rows due to its thinner teeth. Besides, since the crusher claw has 2 different tooth patterns, it is interesting to verify whether the combination of these 2 shapes will perform better than individual ones, which is *crusher*_*com* in Figure 8B.

 Based on recent research published by UC Berkeley (Guo et al., 2017), where 37 finger surface designs were proposed and analyzed, we choose the best performing one as a validation in our study, as shown in Figure 7A. We hope this design can validate whether our designs are competent and worth further exploration. Except for these designs, a pair of bare fingers will also be tested as a baseline to validate whether an external silicone layer is necessary (shown in Figure 7A).

All the silicone materials are Dragon Skin 10 from Smooth-On, with Shore 10A, tensile strength of 475 psi, and maximum elongation of 1,000% at the break to ensure the strength of the contact surface while keeping the fingers flexible. Besides, all the molds are fabricated by 3D printing using resin as material, with an accuracy of 0.1 mm. In addition, the validation uses Dragon Skin 30 with Shore 30A material, which is the best material for this design according to Guo et al. (2017) team. The fabrication process is shown in Figure 7B. The silicone can be tightly wrapped around the links and, thus, be closely attached to the finger.

Before we analyze the result of the experiment, one thing should be mentioned is that the bare fingers are capable of grasping most of the tested objects. Figure 8A is the grasp success rate for the bare fingers on 16 objects; the green line on it represents a success rate of 95%. As it is shown, there are 11 objects higher than 95%, which can be regarded as high reliability and stability. These data can only prove the good performance of the soft, adaptive fingers but is meaningless to show the difference between the finger surface designs. Thus, these objects will be deleted to shortlist the object set to highlight the differences between the designs.

Therefore, in Figure 8B, there are 2 bars for each candidate: the green bar on the left is the mean and variance on all the 16 objects, while another yellow bar on the right is the mean and variance on the shortlisted 5 objects. From the result, we can see that:

• Fingers with contact surface perform better than bare fingers, with 18.2% higher mean and 9.6% lower variance at most, which indicates the necessity of the external contact surface.
• All of our designs perform better than the validation design, with 10.4% higher mean and 5% lower variance at most, which indicates that these bionic designs are competent and worth further exploration.
• The combination of the crusher claw designs performs better than the individual ones, with 3.13% higher mean and 2.4% lower variance at most, which proves that the result of evolution and natural selection is reasonable and convincing.
• Among all the designs, the design imitating the cutter claw performs the best, with a mean of 96.5% and variance of 4.8% on the entire set, while with a mean of 93.1% and variance of 5.1% on the shortlisted set.

Based on this result, we will further explore the design parameters of the cutter design based on its original shape in the following iterations.

### 3.3 Second Iteration: Design Parametric Exploration

**FIGURE 9:**
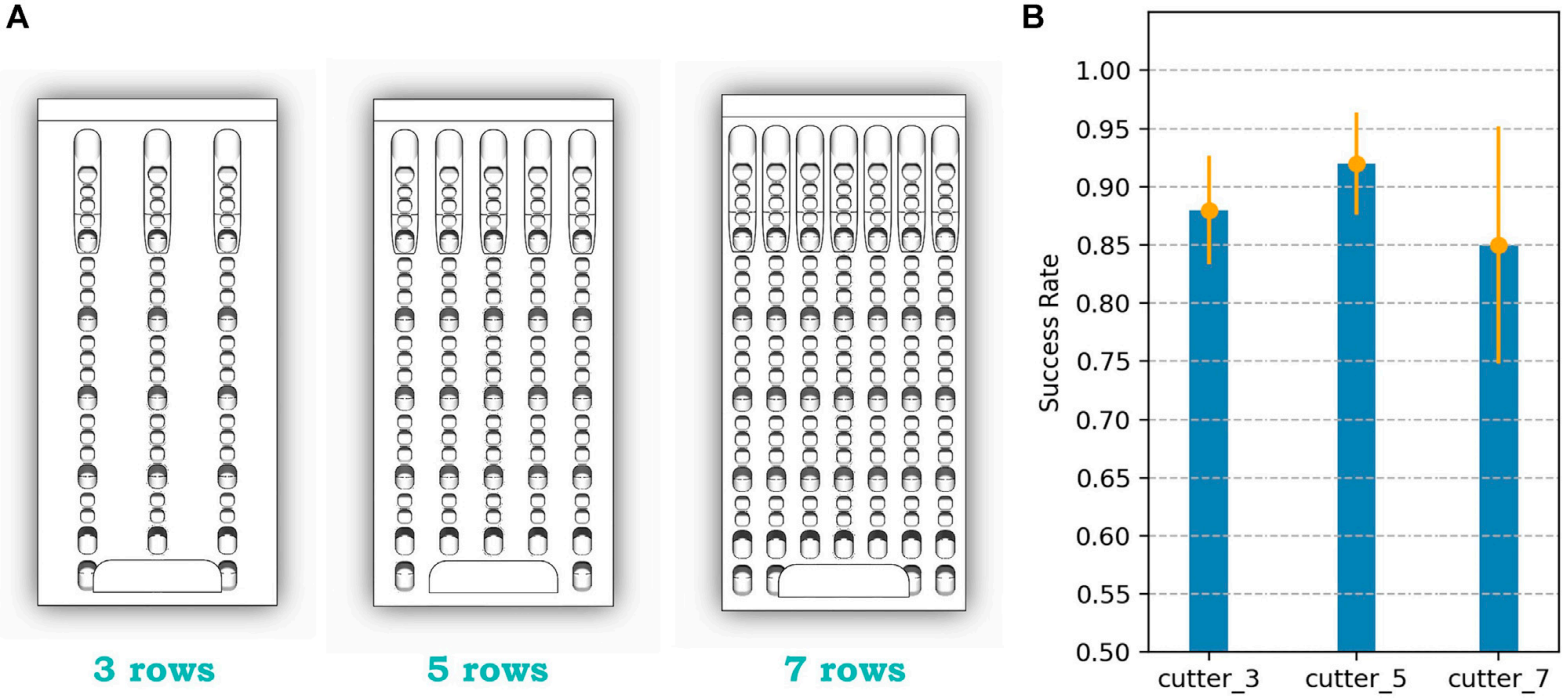
Second iteration designs and their performance, with mean and variance.

In the initial cutter bionic design, we duplicated the row 5 times to fill up the area. However, the density of the row will also be a key factor influencing the grasping performance. Therefore, we expanded our design space by adjusting the density of the rows. Increasing the density forms a 7-row design, and by decreasing the density, it forms a 3-row design. The result is shown in Figure 9. Still, our initiatory design performs the best among the designs in this iteration, with the highest mean of 92% and the lowest variance of 4.4%. Therefore, a row number of 5 will be considered an optimal design parameter to support the following design iteration.

### 3.4 Third Iteration: Bionic Parametric Exploitation

**FIGURE 10:**
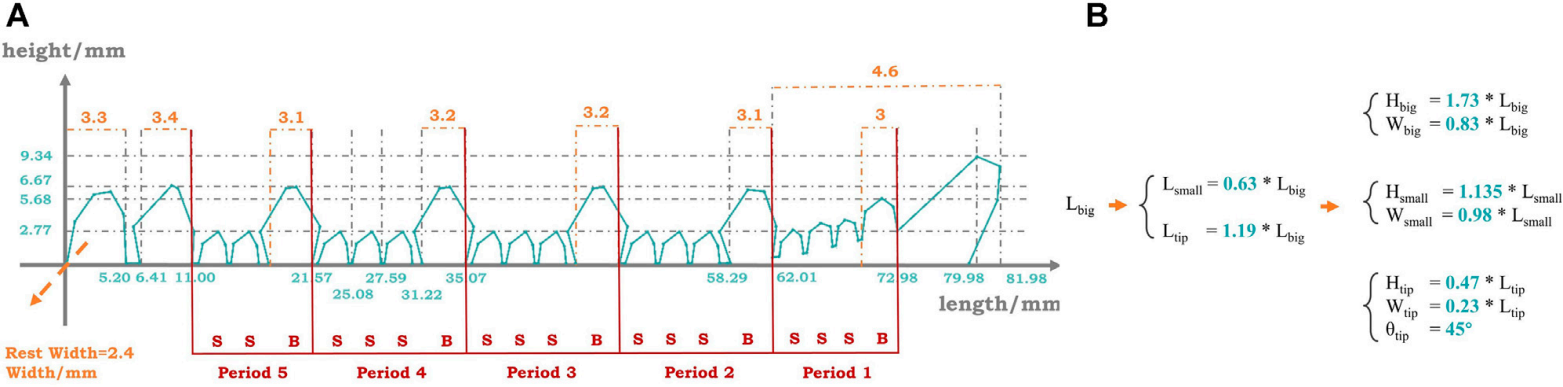
**(A)** Quantitative detail and periodic analysis of the teeth on the cutter. **(B)** Mathematical expression: the quantitative relationship between the teeth on the cutter, with the numeric parameter in blue sampled from a specimen.

The next step is to explore the bionic parameter inside the row. Figure 10A shows the quantitative detail and the analysis of the tooth profiles on the cutter claw of the Boston Lobster. There are mainly 4 types of teeth on the cutter claw: 1) Tip teeth, which is at the distal end of the claw (right side in the figure), bends like the mandibles of a crossbill to lock the object and prevent it from sliding out of the claw. 2) Proximal teeth, which are at the proximal end of the claw (left side in the figure), function as clamping and smashing the object, like the molar of humans. Between the tip tooth and proximal teeth, the teeth are seen to be arranged in a periodic sequence. In each period, there is a 3) big tooth, which is “B” in the figure, followed by a series of 4) small teeth, which is “S” in the figure, and the number of small teeth is approximately 3.

The cutter teeth are developed almost in a linear series, and the order with respect to size corresponds to that of age or time of appearance. The big teeth are the first to emerge. They are set at wide intervals and evenly spaced. After the second molt stage, small teeth will emerge in the intervals of the big teeth, and eventually, at the fourth molt, a single period of 4 teeth is completed. The proximal teeth are the same as the big teeth and also lead their period. However, as the lobster is molting, the small teeth around them will be concentrated and fused by them (Herrick, 1911).

Here, we assumed that the periodic teeth enabled the claw to better fit the complex outer contours so that they could grasp a greater variety of objects. For the general objects in human society, their outer contours are mainly regular and flat. However, most of the objects in nature possess irregular shapes and uneven surfaces, which enlarges the shape complexity and, thus, grasp difficulty. In this way, the grasping tools of some creatures in nature are also irregular, such as the claws of the lobsters in this case.

**FIGURE 11:**
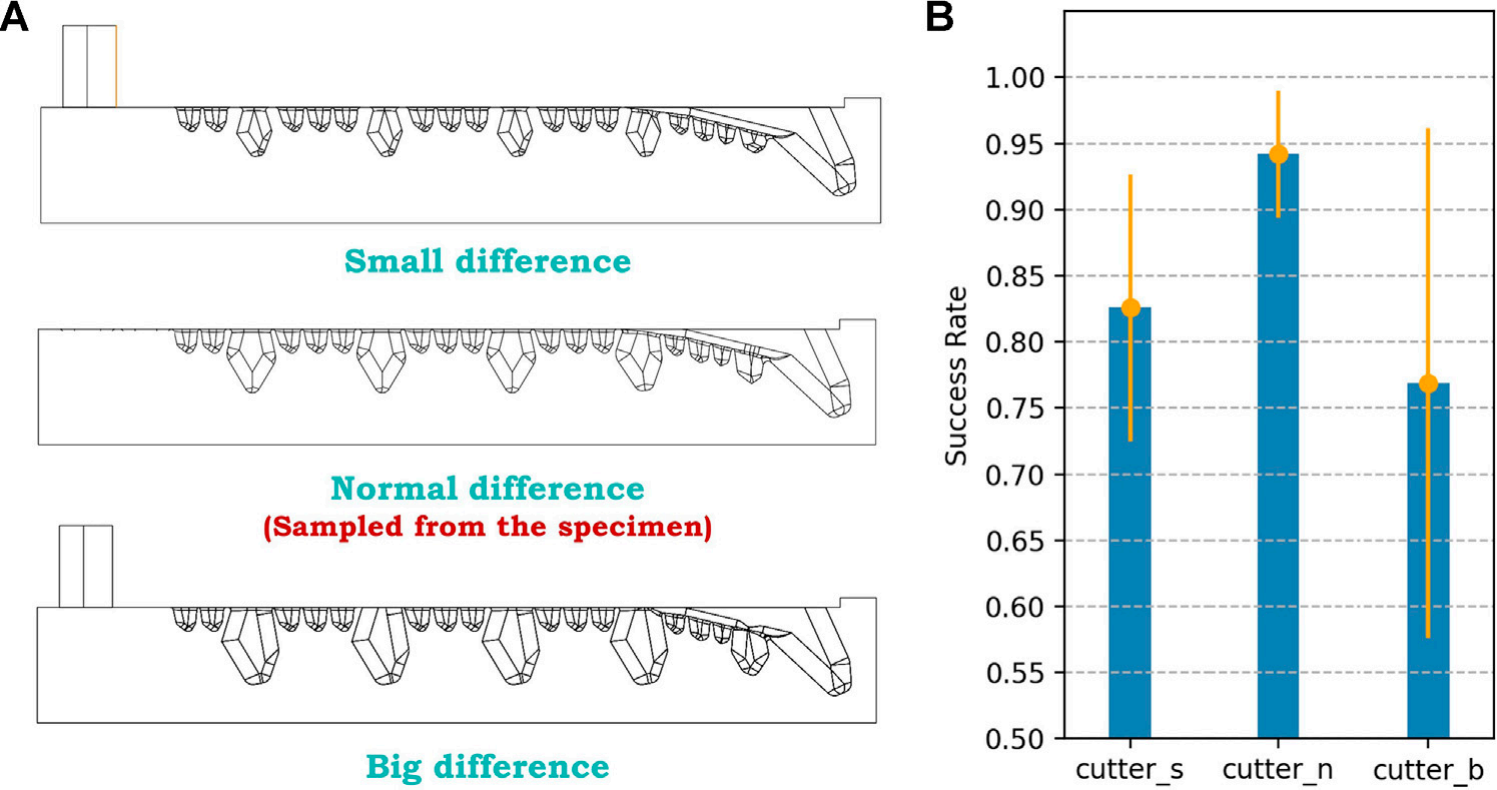
Third iteration bionic parametric exploration designs and their performance, with mean and variance.

Given this assumption, we hoped to find the relationship in size between the big tooth and the small tooth to obtain an optimal parameter ratio. There are mainly 3 variables to decide the size of a tooth, which are length (L), width (W), and height (H). Based on a Boston Lobster specimen, we came up with a group of mathematical expressions on size relationship, as shown in Figure 10B. By these expressions, given a certain length of the big tooth as input, we can get all the sizes of the teeth, which can be further adjusted and applied to other robotic fingers. By adjusting the observed parameter, we can alter the difference in size between the big tooth and the small tooth. Therefore, as shown in Figure 11, additional 2 designs are formed, along with their experiment result. *cutter_n* represents the normal difference between big teeth and small teeth, which is sampled from the claw directly; *cutter_s* represents the small difference, with about 1/3 smaller than the normal in bulk; and *cutter_b* represents the big difference, with about 1/3 larger in bulk.

According to the result, the initial design still performs the best, with the highest mean of 94.2% and the lowest variance of 4.8%. The gap of this iteration between the designs is more significant than all the iterations above, with a gap in mean up to 17.4% and a gap in variance up to 14.4%. The small difference design performs worse than the validation design in the first iteration, and the big difference design performs even worse than the bare finger, with approximately the same mean but a 4.5% higher variance. Therefore, we considered the original bionic parameters on the Boston Lobster specimen to be the optimal solutions, which means the initial cutter design is the optimal finger surface design *d*∗ after the 3-stage design iteration process.

### 3.5 Grasping Performance Evaluation: On Land Versus Underwater

Now that the optimal design has been selected through 3 design iterations and experiments held on land, we wonder whether the lobster-inspired finger surface design can still benefit robotic grasping in the underwater scenario since the contact mechanics becomes challenging and complicated when water is involved. Therefore, we set up a 53 cm × 42 cm in length and width blanket, and filled it with 15 cm in depth of water to simulate an underwater scenario. This experiment follows the same policy as on land and that all 16 objects will be grasped, each object will be grasped 80 times for each design, and the air pressure for the gripper is still 1.0 MPa as on land.

**FIGURE 12:**
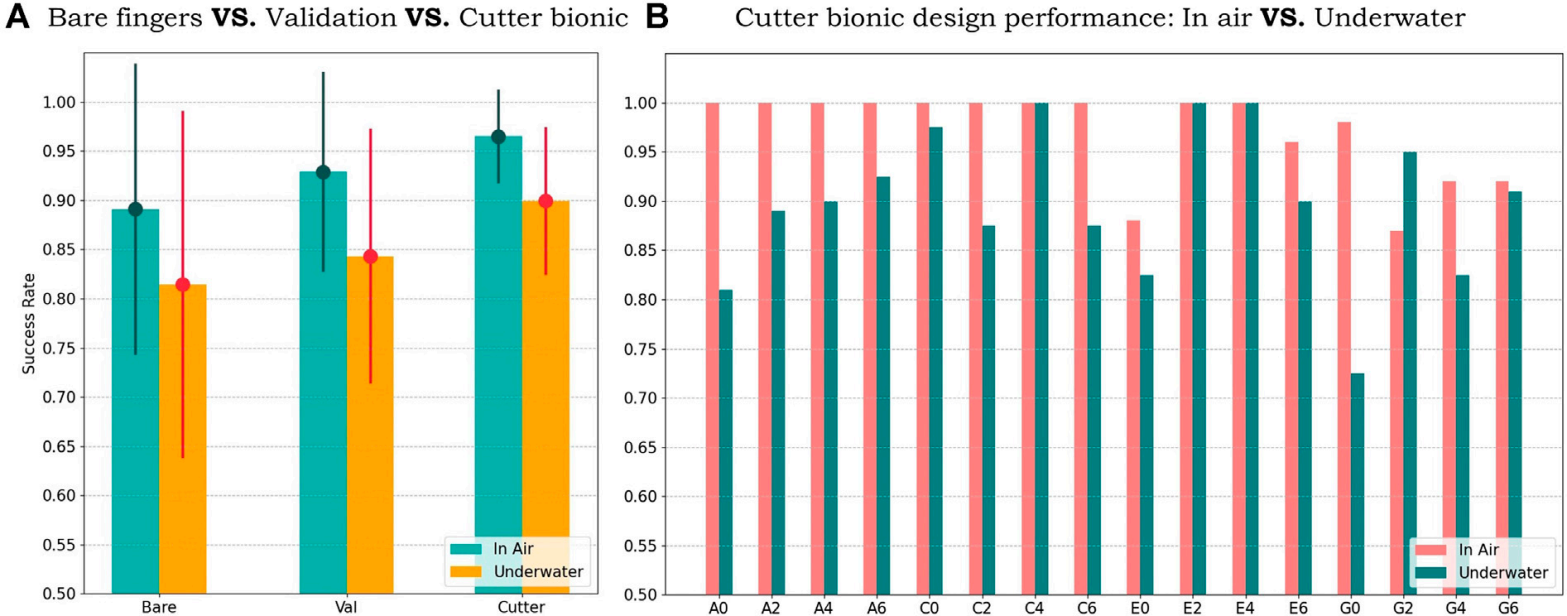
**(A)** The performances of the bare fingers, validation design, and optimal cutter bionic design on 16 objects in air (on land) and underwater, with mean and variance. **(B)** Optimal cutter bionic design performance detail on 16 objects, in air (on land) and underwater.

The Figure 12A shows the grasping success rate result of the 3 candidates, which are bare fingers, validation design, and the optimal cutter bionic design. Both of them performed worse than in the on-land scenario, with 7.71%, 8.57%, and 6.57% decrease, respectively. However, the underwater result shows the same feature as in the on-land case:

• Fingers with simple contact surface pattern, which is the validation design, performs better than bare fingers, with 2.9% higher mean and 4.7% lower variance, also indicating the necessity of the external contact surface in the underwater scenario.
• The optimal cutter bionic design still shows the best grasping performance: a mean of 89.90% and a variance of 7.51%, with 8.49% higher mean and 10.14% lower variance than bare fingers, and 5.59% higher mean and 5.44% lower variance than the validation design.

Figure 12B shows the grasping success rate comparison for the optimal cutter bionic design on all the 16 objects in air (on land) and underwater, with 1 object improved: 8% on G2; 3 unchanged: both performed at 100%; 12 declined: 25.5% the most on G0.

## 4 Discussion

### 4.1 Analysis on the Result of 3 Iteration Designs

In the first iteration, a raw imitation of the Boston Lobster specimen, the cutter design performs the best. History has shown that the cutter represents an original or an older type and that the crusher was later developed from it. These 2 claws show no difference until the seventh or succeeding molt stage in the growth of a lobster, with the teeth rounding and blunting, particularly at the proximal end. Then, it follows a characteristic process of concentration and fusion in the spines of the future crusher claw; the crushing tubercle is, thus, formed (Herrick, 1911).

The result can be explained in that the cutter in this primitive type is already capable of the fundamental manipulation tasks encountered by the lobster. However, as the competition for survival has become fierce, more challenging tasks, such as defense and predation, require advanced weapons and tools so that the crusher occurs. Actually, in all measurements except length, the crusher greatly exceeds its fellow, being one-third broader, weighing twice as much (in the dry shell), and having more than double the cubic capacity (Herrick, 1911). However, “in animals of adult size, the slenderer cutter has often a slight advantage in length over the more powerful crusher,” according to Francis H. Herrick, professor of Biology. In this way, the cutter is assumed to be more capable of grasping tasks than the crusher. Therefore, our first stage experiment result is consistent with the biology research.

The second iteration exploring the design parameters shows that the 5-row design performs better than the 3-row and 7-row designs. This result indicates that neither a dense surface nor a sparse surface benefits the grasping performance well. A balancing point should be found between too dense and too sparse to suit the objects in various shapes, sizes, and textures that will be encountered.

In the third iteration exploiting the bionic parameters, the result shows that the initial cutter bionic design performs the best. The development of lobster can explain this result. As mentioned previously, a 4-tooth period is completed after the fourth molt stage, but the process does not always stop there, as in the seventh or eighth molt stage, with a small probability, more teeth might emerge (Herrick, 1911). There will be more molt stages to come. However, most periods found on the cutter of lobster are of the “traditional” 4 teeth, which can be regarded as the result of evolution and natural selection that this pattern and the size of the teeth are already enough for the manipulation tasks in daily life. Given this assumption and the third stage experiment result, the pattern, relationship between the big and small teeth, and other bionic parameters are already optimal and converged on an adult lobster.

### 4.2 Analysis on the Difference Between On-Land and Underwater Grasping Performance

As mentioned in the *Results* section, all the candidates were performing worse underwater than on land. Bare fingers have a 7.71% reduced mean and 2.86% increased variance, validation design has an 8.57% reduced mean and 2.77% increased variance, and cutter design has a 6.57% reduced mean and 2.74% increased variance. As we record and analyze the failure cases, we believe water is primarily responsible for the decline in grasping performance.

There are 2 typical grasping failures both on land and underwater given an appropriate gripper:

• Collision: The gripper collides with the object during the approach, causes the object to displace from its observed position and change its orientation so that the gripper fails to grasp the object using a piece of expired location information.
• Sliding: The gripper touches the object successfully, but not tightly enough (often occurs when the orientation of the object is hard to grasp, for example, a phone lies flat on the table). This will cause the force provided by the fingers smaller than the gravity of the object; thus, the object will slide from the gripper and drop.

Given these 2 typical failure modes, water can influence them differently:

• When the object sinks in the water, it is provided with buoyancy, which can offset the gravity. Therefore, the object can be easily influenced by the hydrodynamic force. For example, as the gripper touches the object underwater with the same velocity and force, the object will float far more farther than in air. In this way, any collision that occurs underwater will cause the object to displace far from its original position, which leads to a grasp failure using a piece of old location information. While on land, the displacements caused by a collision are often acceptable for our gripper.
• As the object is covered with water, water becomes an inter-medium between the object surface and the finger surface, and thus, it will decrease the friction force between them, compared with air. Therefore, the friction force applied by the fingers underwater can be smaller than in air. What is more, the water attached to the object will increase its weight, which further increases the difficulty of grasping.

In the on-land scenario, after the gripper collides with the object to cause it to displace, or the location observed by the system has a relatively large error, sometimes the gripper is still able to grasp the object successfully, thanks to the additional friction force provided by the silicone layer. While in the underwater scenario, the force provided by the fingers is no longer enough to deal with the challenges, so that the objects are more likely to slide from the gripper.

We came up with some possible solutions:

• Enlarging the size of the gripper: To avoid the collision between the gripper and the object during the approach, the space between the fingers on the gripper should be big enough.
• Stronger finger material: The material of our current soft finger is too soft that it will severely deform given an air pressure of over 1.0 MPa. If the finger is built by a stronger material, it can bear and also provide a bigger force.
• Larger air pressure for the gripper: Currently, we use 1.0 MPa air pressure to close the gripper, which is limited by the material of the finger. If the finger permits, bigger air pressure can help the gripper to grasp the object more tightly to prevent sliding.
• Hydrophobic treatment of the finger surface: Using certain hydrophobic chemicals, the finger surface can suffer less from the water and, thus, perform better.

## 5 Final Remarks

### 5.1 Conclusion

In this paper, we put up a total of 7 contact surface designs inspired by the claws of the Boston Lobster in a 3-stage design iteration process to enhance the stability and reliability of robotic grasping. To verify their performance, we built up a robotic grasping platform and introduced a test set of 16 objects from EGAD to be grasped. Eventually, after more than 14,000 grasp attempts, the result proves that compared with the bare finger and validation design, the best-performing design, the initial cutter bionic design, is of remarkable benefit to the grasping performance with 18.2% and 10.4% success rate improvement at most, respectively. It proves that an external silicone contact surface with peculiar patterns benefits the reliability and stability of robotic grasping. Besides, in the underwater grasping performance, the cutter design still shows good stability and reliability on object manipulation compared with the bare fingers and validation design, proving its capability and competence in an amphibious environment. Therefore, we believe it has promising potential and can contribute to future research in enhanced underwater grasping.

What is more, it is interesting to see that the experiment result is consistent with bioresearch on lobsters:

• Our result shows that the cutter bionic design performs better than all of the crusher bionic designs, with the fact that the cutter is the primitive grasping tool for lobsters, and the crusher is developed from it for advanced requirements.
• The cutter design with bionic parameters sampled from the specimen performs the best, with the fact that lobsters possess fully developed cutter about halfway through their molt stage, which indicated that the size, shape, texture, and pattern of the cutter have already converged to optimal through the selection of nature and barely changed for the rest of their lives.

The consistency with the bioresearch further justifies our results and also illustrates the value of bionics that the answers we have been looking for might already exist in the creatures in nature.

### 5.2 Limitations and Future Work

In this paper, we discovered some limitations and hoped to improve them in the future:

• The designs in the second and third iterations both fail to improve the grasping performance, which indicates our design method should be further optimized. In the future, we hope to use computational method to analyze the arrangements of the teeth profiles on the lobster claws and generate a series of designs based on the arrangements automatically to enlarge the design pool.
• In our previous research, we have successfully added optical fibers to the finger and used the data collected from the fibers to predict force, torque, and contact on the finger based on machine learning methods (Yang et al., 2021). In the future, we will install these fibers into the finger surface so that the surface itself can “feel” the object. In this way, we can use these physical indicators to explain how a well-performing design can influence grasping specifically.
• We selected the optimal design empirically based on the success rate of grasping, which is a statistical indicator, without further recording and analyzing the physical indicators on why the optimal design performs the best. In the future, we expect to set up a mathematical expression, with physical indicators, such as contact area or friction force provided by the contact surface measured by the above method, as input, and with possible grasping performance as output. It aims to predict whether a finger surface design is beneficial or not with a considerable load of testing so that we can explore more possible designs.
• We only tested our design inside a blanket. In the future, we hope to install our finger surface design to the gripper on the diving robot and use them to grasp objects truly underwater, such as the shells under the water and the rubbish floating on the water. In addition, only a superficial attempt is made in revealing the secret of the grasping ability of the lobster:
• In the design that mimics the cutter of the Boston Lobster, we used the pentagon to simulate the tooth shape. However, whether other shapes can better present the tooth has not been tested, which remains a variable influencing the grasping performance.
• Besides, the size of the tip tooth also deserves further discussion. Acting as a hook on the distal end of the claw, whether a larger tip or a smaller one can better benefit robotic grasping still needs to be proved.
• The design is not precisely consistent with the original claw. In the cutter design, we arranged the teeth to be linear and symmetrical in the center. However, the authentic teeth are arranged in 2 lines on the cutter, with big teeth and small teeth in different lines. Therefore, thanks to this arrangement, the close pose of the claw is by overlapping its teeth on the upper and lower jaws, but not interlocking.
• Last but not the least, the actual 4 teeth period of the cutter is “1 3 2 3,” in which the numerics represent the size order of the teeth. For example, the big tooth is the order “1.” However, we use only one type, the “small tooth,” to represent the order “2” and order “3” teeth, which is not consistent with reality.

Deeper exploration is still required to reveal the secret of the grasping ability for the Boston Lobster, other lobsters, and even all the crustaceans. There is still more to learn from nature.

## Data Availability

The raw data supporting the conclusion of this article will be made available by the authors without undue reservation.
